# Early Catastrophic Failure of Highly-Cross-Linked Polyethylene Liner Due to Acetabular Shell Screw Placement: A Case Report

**DOI:** 10.7759/cureus.115743

**Published:** 2026-09-03

**Authors:** David Dallas-Orr, Gregory J Harbison, Samuel K Simister, Shannon Tse, John Meehan

**Affiliations:** 1 Orthopaedic Surgery, University of California Davis Health, Sacramento, USA

**Keywords:** acetabular liner failure, acetabular screw protrusion, hip joint, polyethylene liner, revision arthroplasty

## Abstract

Highly cross-linked polyethylene (HXLPE) has become the standard acetabular liner material in total hip arthroplasty (THA) because it has markedly reduced wear-related osteolysis and aseptic loosening. However, cross-linking also reduces resistance to fatigue and crack propagation, which may lead to failure in areas of concentrated stress. Acetabular liner failure remains rare and most commonly occurs following trauma.

We report an atraumatic early catastrophic failure of an HXLPE acetabular liner in a 62-year-old woman, 20 months after primary THA at an outside institution. She presented with groin and lateral hip pain, joint squeaking, and an inability to flex the hip past 90 degrees. Radiographs demonstrated eccentric positioning of the femoral head within the acetabular shell and displacement of the acetabular locking ring. During revision, the liner was found fractured into multiple fragments with surrounding metallosis. An eccentrically placed acetabular shell screw protruded into the inner surface of the shell, and the location of the screw head corresponded with backside liner wear. The rim screw was removed and the liner was exchanged. At telephone follow-up over eight years later, the patient reported ambulating without difficulty and denied pain or subsequent revision.

This case describes a preventable, technique-dependent mechanism of early HXLPE liner failure and supports routinely confirming flush seating of the acetabular screw before liner insertion during THA.

## Introduction

Total hip arthroplasty (THA) is a highly effective surgical procedure for the management of various hip pathologies including end-stage hip osteoarthritis, with excellent patient reported outcomes and long-term implant survivorship. Historically, non-cross-linked ultra-high-molecular-weight polyethylene (UHMWPE) was favored for the acetabular liner component in THA due to its low friction and wear-resistant properties [1,2]. However, the most common cause of late revision THA with a metal or ceramic head on a conventional UHMWPE liner was aseptic loosening driven by osteolysis resulting from particulate wear debris [3,4].

The development of highly cross-linked polyethylene (HXLPE) liners has markedly reduced THA revision rates related to osteolysis and aseptic loosening [5-8]. HXLPE is manufactured utilizing sequential irradiation and annealing processes that enhance wear resistance by forming carbon-carbon bonds between polymer chains and reducing oxidation and free radical formation [9]. Although these manufacturing steps improve wear performance, they may also reduce resistance to fatigue crack propagation and fracture under concentrated or repetitive stress [10,11]. Accordingly, while catastrophic liner failure remains uncommon, accounting for less than 1% of early revision THAs [12], early mechanical failure has been reported in high-stress regions such as the liner rim, locking mechanism, or areas exposed to impingement or localized point-loading [13,14].

HXLPE liners are widely used in modern THA. Therefore, understanding the risk of fatigue failure and minimizing technical factors that concentrate stress on the material are important. Previously reported failure mechanisms have largely been related to the implant or component malposition, which may be difficult for the surgeon to address during the index operation. In this case, the stress riser resulted from a single modifiable technical step, the seating of an acetabular shell screw. This mechanism has been described with a ceramic liner [15] but is rarely reported with HXLPE. This report defines the angular tolerance needed to avoid this failure and identifies a simple intraoperative check to confirm appropriate screw seating.

## Case presentation

Clinical findings and diagnostic imaging 

A 62-year-old female with a medical history of fibromyalgia, lower back pain, and BMI of 39.7 was referred to our institution in 2015 with complaints of groin and lateral hip pain following a primary left THA performed at an outside hospital 20 months earlier. Three months prior to being seen at our institution, she experienced an acute worsening of pain without trauma, characterized by sharp pain and an inability to bear weight. She was placed on bed rest for one month by the direction of an outside surgeon and later resumed ambulation with assistance. Upon presentation to our clinic, the patient described a constant, sharp pain that began acutely after flexing her hip. She also reported associated squeaking in the joint and an inability to flex her hip past 90 degrees.

On physical examination, the patient was unable to flex her left hip past 90 degrees, with pain limiting both internal and external rotation. Her gait was antalgic, and she had a positive Trendelenburg sign. Radiographs of the left hip showed eccentric positioning of the femoral head within the acetabular shell, with cephalad displacement of the prosthetic femoral head and osteolysis at the lateral aspect of the acetabular component (Figures 1, 2). The femoral component was in a neutral position. Additionally, radiographs showed displacement of the acetabular locking ring. Failure or dislodgement of the polyethylene acetabular liner was suspected, and surgical revision was planned.

**Figure 1:**
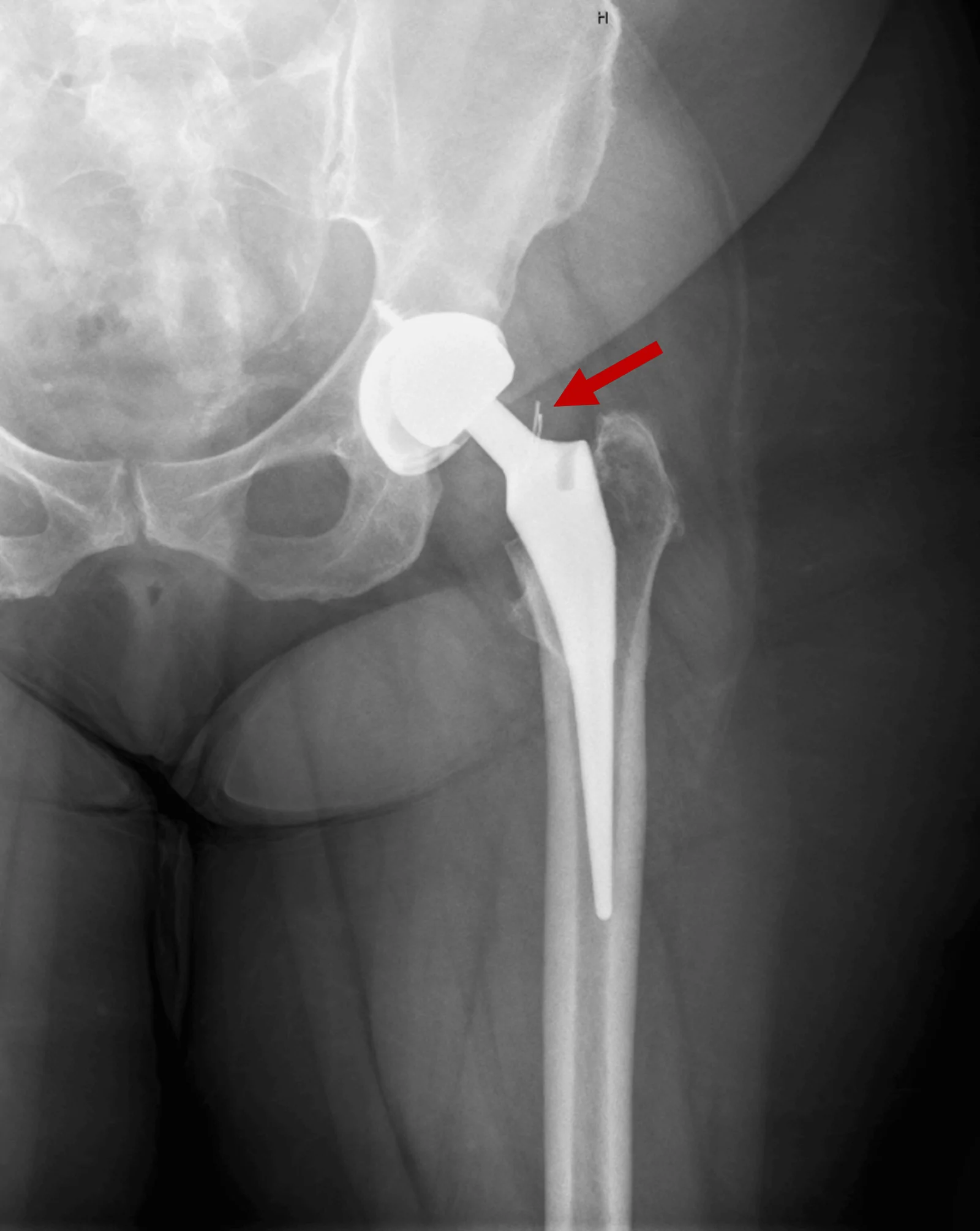
Anterior posterior radiograph of the left hip showing eccentric positioning of the femoral head implant within the acetabular shell, and a displaced RingLoc mechanism (red arrow) around the femoral stem

**Figure 2:**
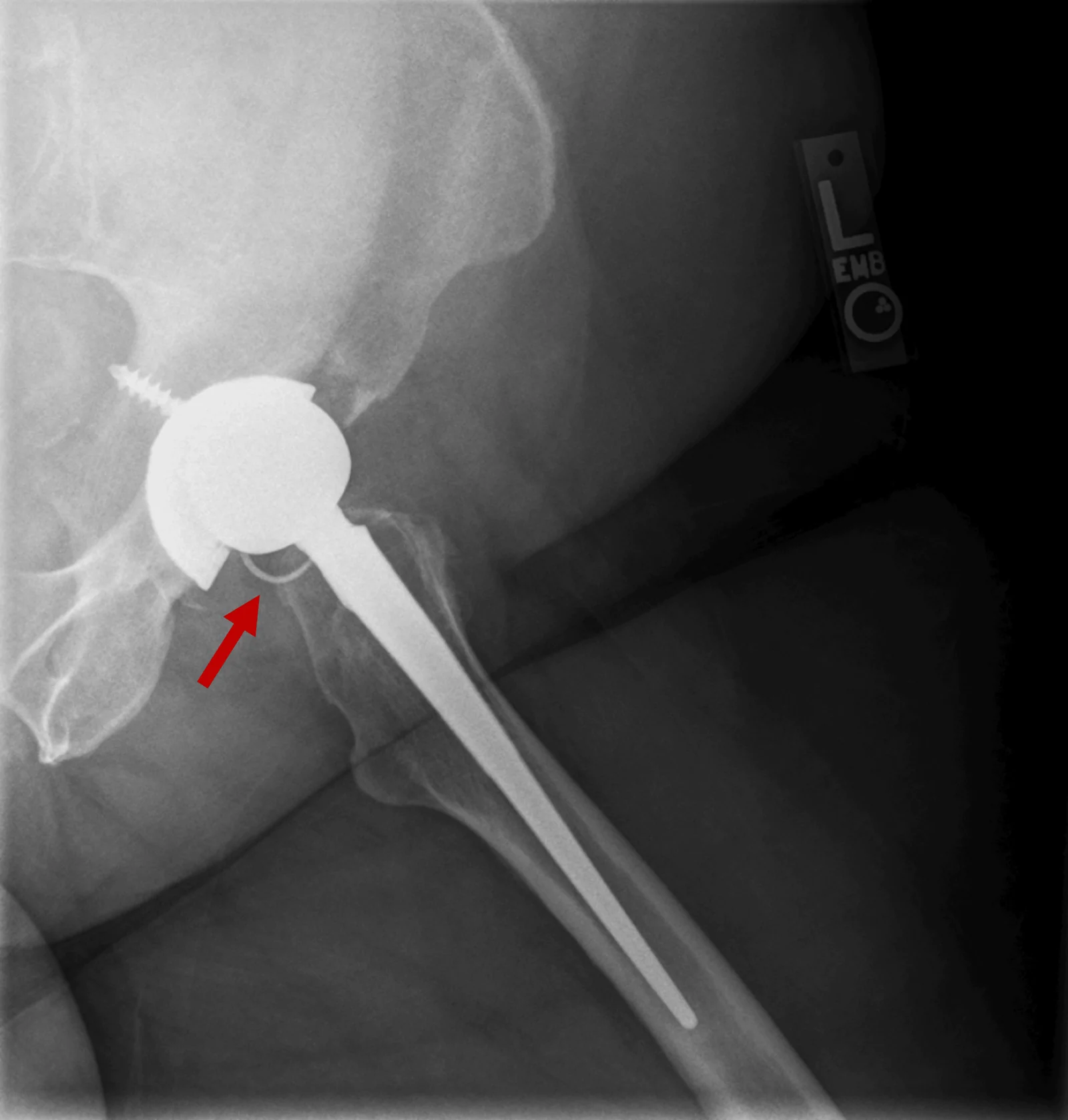
Frog leg lateral radiograph of the left hip confirming displacement of the RingLoc mechanism (red arrow) without evidence of periprosthetic fracture

Records from the outside hospital indicated the primary THA was performed via a posterior lateral approach. The prior non-cemented implants consisted of a femoral stem (size 6 x 132) with a cobalt-chromium head (36 mm, +3 mm offset), a RingLoc acetabular shell with an HXLPE liner, and a single acetabular shell screw (Zimmer Biomet, USA) placed in the posterior superior quadrant.

Surgical intervention 

A posterior lateral approach was used with the patient in lateral decubitus position. Intraoperatively, synovialized membrane was found in the subcutaneous tissues superficial to the deep fascial layer, suggesting prior seroma formation. A dark, black fluid, consistent with metallosis, was aspirated from the joint space, and the periacetabular synovial tissues were stained with metallosis.

The HXLPE acetabular shell liner was fractured into multiple pieces (Figure 3) with the RingLoc metal mechanism (Zimmer Biomet, USA) displaced into the surrounding soft tissues. The acetabular dome screw was found to be eccentrically placed in the shell hole, protruding into the inner surface of the acetabular shell. Eccentric wear present on the backside of the liner in continuum with the margins of the fracture fragments corresponded with the location of the prominent screw. The screw was removed, and both the acetabular and femoral components were confirmed to be well-fixed both visually and radiographically. The acetabular component was appropriately positioned regarding inclination and anteversion. The femoral component was appropriately positioned regarding version.

**Figure 3:**
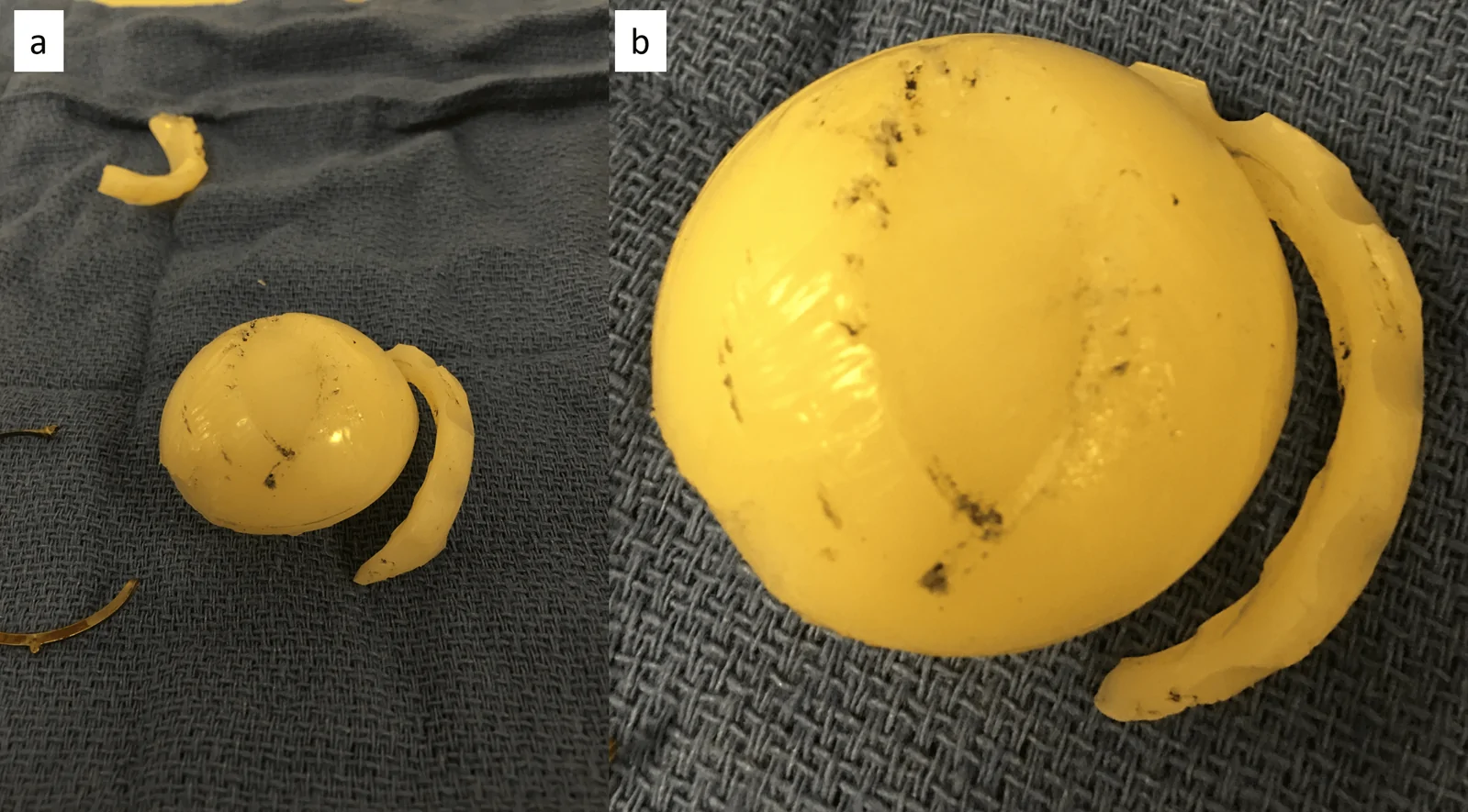
(a) Fragmented HXLPE acetabular shell liner with 3 large fragments and the RingLoc mechanism; (b) Close-up image showing significant wear and metallosis staining

Due to the well-fixed and well-positioned existing components, a constrained liner was selected for revision. A series 23 constrained liner, RingLoc mechanism, and 36 mm neutral Freedom head (Zimmer Biomet, USA) were placed. The components were confirmed to be well-seated, and the hip was concentrically reduced. Intraoperative radiographs confirmed reduction and appropriate placement of the components. A post-operative radiograph showed anatomic alignment of the implants without evidence of hardware complications (Figure 4). The patient was given instructions to bear weight as tolerated using a front wheeled walker for six weeks. She ambulated with the physical therapist prior to being discharged home.

**Figure 4:**
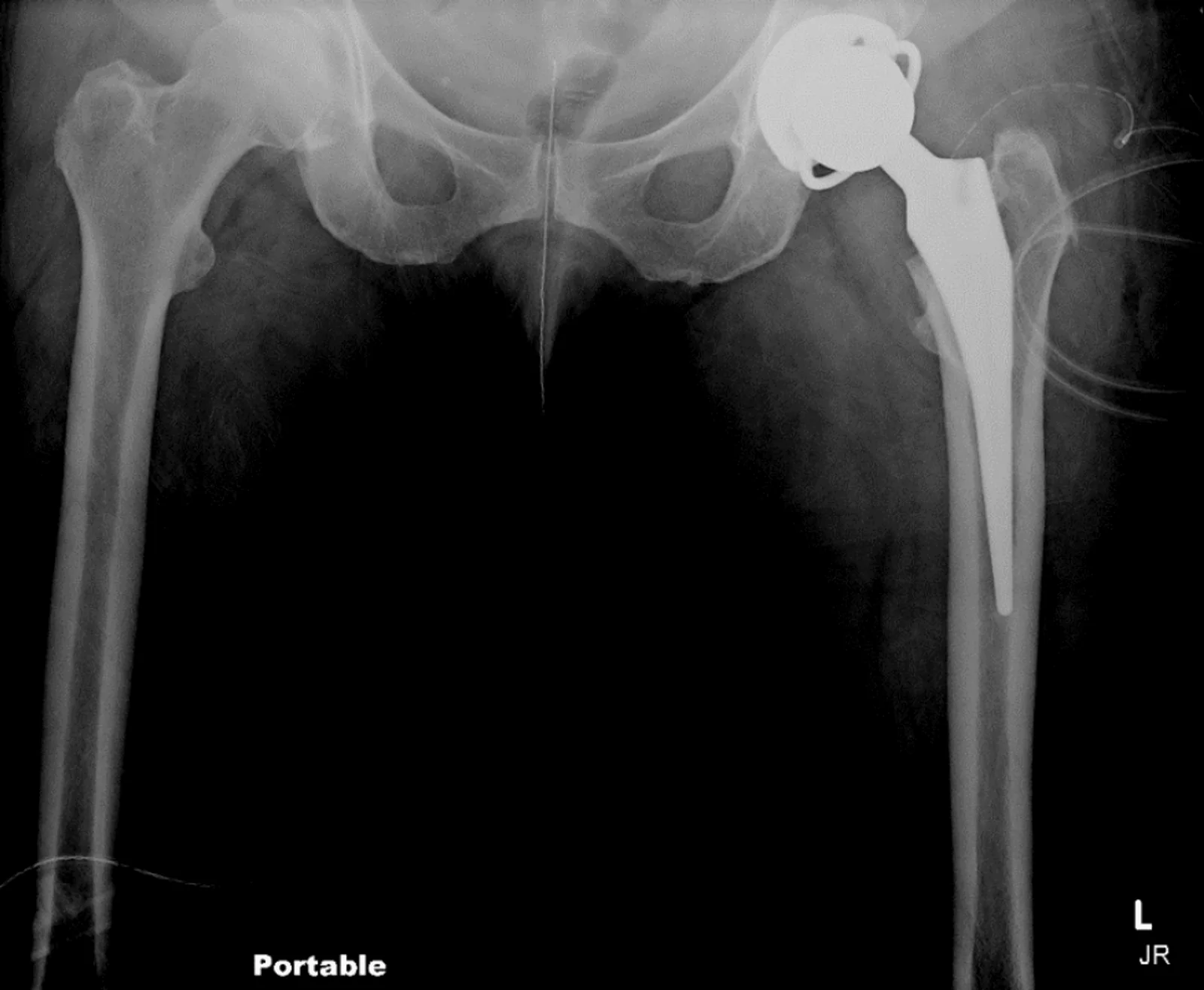
Post-operative portable anterior posterior pelvic radiograph showing removal of the acetabular screw, concentric positioning of the femoral head within the acetabular shell and a replaced RingLoc mechanism. No periprosthetic fracture was observed.

Follow-up and outcomes 

At the two-week follow-up, physical examination revealed a well healing skin incision, and radiographs did not show signs of loosening or failure. The patient was able to flex her hip to 90° without pain. At six weeks, the patient continued to use a front wheeled walker, and a repeat pelvic radiograph was unchanged without signs of osteolysis, loosening or implant failure (Figure 5). Over eight years later, at an out-of-state hospital, the patient received a surgical evaluation for pain in her contralateral hip. At this time, the patient followed up with our clinic over the phone and reported ambulating well on her left leg. She denied having any pain in her left leg and denied having any complications or revision surgery on her left hip since her six-week follow-up appointment.

**Figure 5:**
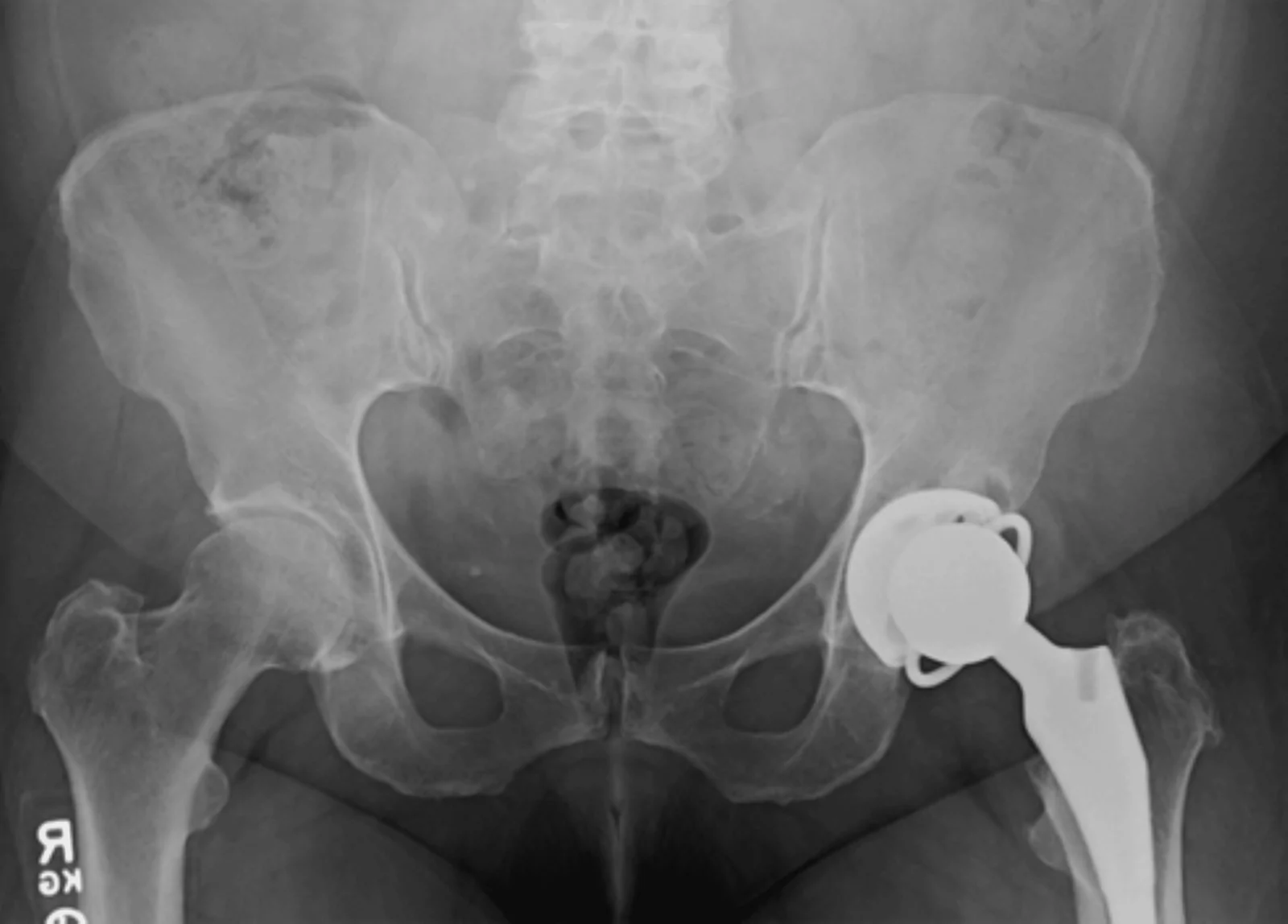
Anterior posterior pelvic radiograph at six-week follow-up, without evidence of periprosthetic fracture or dislocation

## Discussion

Overall revision rates have ranged from 0 to 8.6%, while the revision rates due to polyethylene wear or osteolysis range from 0 to 0.01% [16]. The development and widespread utilization of HXLPE liners has contributed to the reduction in revision rates [17,18]. However, the improved wear properties of HXLPE come with a tradeoff of reduced fatigue and fracture resistance [17,19]. Significant advancements in material science and manufacturing including vitamin E treatment, irradiation methods, and post-radiation thermal treatments, have helped address these materials characteristics, but continued post-market surveillance and evaluation of long-term data is needed to evaluate their superiority [17].

Our case report details the catastrophic failure of an HXLPE liner due to eccentric and prominent placement of an acetabular shell rim screw and highlights the importance of surgical technique in THA to promote survivorship. The screw trajectory must be within a pre-specified range which varies based on the acetabular shell and screw choice. For the RingLoc acetabular shell and rim screw in this case, the allowed range is +10 to -10 degrees from the trajectory of the screw hole of the acetabular shell. This leaves the orthopedic surgeon with a 20-degree target for screw placement (Figure 6). Deviations outside this range will lead to the screw head sitting prominently above the inner surface of the acetabular shell. In this case, the backside of the HXLPE liner was subjected to localized stress due to the prominence of the screw head. A similar early-failure mechanism was observed by Lee et al. in a ceramic-on-ceramic head-liner combination due to prominent acetabular screw placement [15]. Backside wear may also be implicated in higher rates of periacetabular osteolysis observed in THA utilizing acetabular screws compared to cementless components fitted without screws [20].

**Figure 6:**
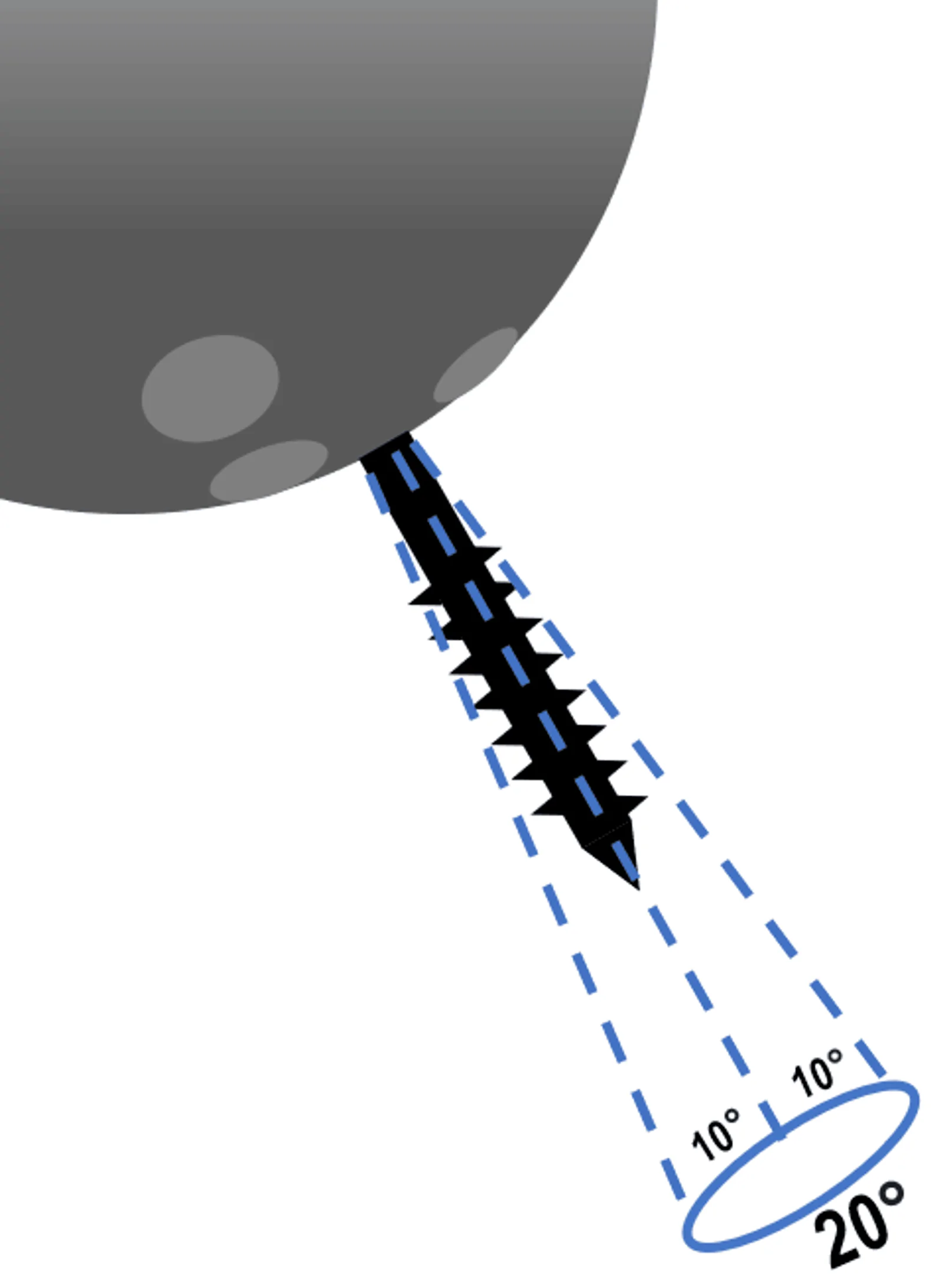
Surgical technique diagram for acetabular rim screw placement, annotated with the –10 to +10 range for the RingLoc implant system The figure has been created by Dr. David Dallas-Orr on Microsoft PowerPoint.

In summary, avoidance of acetabular screw malposition can be accomplished through adherence to appropriate surgical technique. We recommend utilization of a variable angle drill instead of a fixed angle drill to avoid screw angulation outside of the accepted margins. Similarly, the drill guide must be fully seated in the shell’s screw counterbore to create a pilot hole within the limits of angulation trajectory. Additionally, prior to insertion of the liner, we recommend confirming flush positioning of the acetabular screw with visual and tactile inspection.

## Conclusions

In conclusion, this case report demonstrates a rare occurrence of an atraumatic failure of a HXLPE acetabular cup liner due to wear and localized stress from a prominent acetabular rim screw. Additionally, it contributes to the literature for the evaluation of the failure mechanisms of HXLPE liners. Lastly, it emphasizes the importance of proper surgical technique during the placement of acetabular screws.
